# Progress on encapsulation and entrapment of enzymes in electrospun nanofibers

**DOI:** 10.1007/s00449-025-03244-z

**Published:** 2025-10-21

**Authors:** Ke Xin Eer, Roshanida A. Rahman, Nur Aizura Mat Alewi

**Affiliations:** 1 Department of Bioprocess and Polymer Engineering, Faculty of Chemical and Energy Engineering, Universiti Teknologi Malaysia, 81310 Skudai, Johor Malaysia; 2 Innovation Centre in Agritechnology for Advanced Bioprocessing, Universiti Teknologi Malaysia, 84600 Pagoh, Johor Malaysia

**Keywords:** Enzyme immobilisation, Entrapment, Encapsulation, Electrospinning, Nanofibers

## Abstract

Electrospun nanofibers have attracted significant interest due to their high surface area-to-volume ratio, porosity, interconnected voids, and advantageous mechanical, chemical, and physical properties. Enzymes, known for its exceptional catalytic properties, are promising candidates for various industrial applications. However, the use of free enzymes is limited by challenges such as poor recyclability and susceptibility to environmental factors. Immobilization techniques offer a viable solution by enhancing the stability and activity of enzymes. This review compares four enzyme immobilization methods to identify the most effective strategy and focuses on the various approaches to optimize electrospinning methods, as well as parameters to maximize enzyme loading, activity retention, and stability. Among the various immobilization methods, entrapment and encapsulation of enzymes within electrospun nanofibers have garnered significant attention in recent years. The review discusses the applications and challenges associated with enzyme entrapment and encapsulation using electrospinning. Overall, advancements in electrospun nanofibers with encapsulated or entrapped enzymes highlight their potential as robust, efficient, and sustainable platforms for biosensors, therapeutics, antimicrobial applications, smart textiles, as well as food and wastewater treatment processes. Subsequently, future research should focus on scalable electrospinning processes, the development of eco-friendly materials, long-term enzyme stability, multi-enzyme systems, and a deeper mechanistic understanding to further enhance performance and safety.

**Graphical Abstract:**

Fig. a

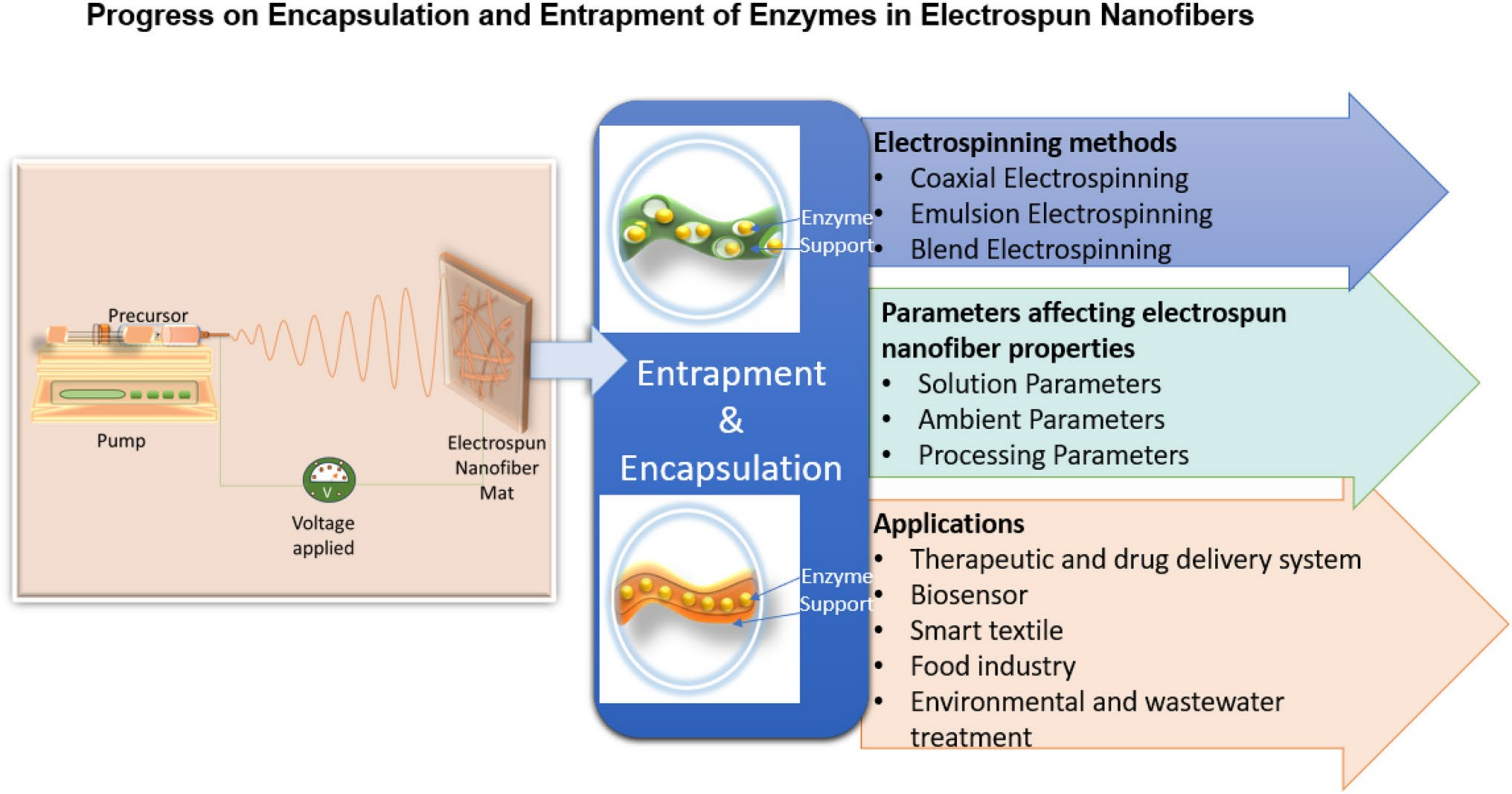

## Introduction

### Electrospun nanofibers

Nanofibers, a type of support characterized by their nanoscale external dimensions, were originally classified as fibers with diameters less than 100 nm. However, their definition has since expanded to generally include fibers with diameters below 1000 nm. Nanofibers can be synthesized using a variety of methods, such as template synthesis [1], solution blow spinning [2, 3], dry-spinning [4, 5], melt spinning [6], wet-spinning [7], force spinning [8], and electrospinning [9]. Among these methods, electrospinning stands out as the the most widely used and efficient method for synthesizing electrospun nanofibers [10].

### Degradation and toxicity of electrospun nanofibers

Electrospun nanofibers have attracted growing research interest across a wide range of applications including biomedical engineering, biosensors, environmental technologies, and the food industry. For instance, Rana et al. [11] demonstrated that electrospun poly (vinylidene difluoride)/nitrogen doped rGO (reduce graphene oxide) nanofibers (~220 nm) enhance triboelectric nanogenerator performance, generating 368 V, 35 μA, and 282.8 μW/cm^2^, making them ideal for sensors applications. Similarly, electrospun polylactic acid (PLA)-zein nanofibers membrane achieved 98.14% PM2.5 and 97.39% PM10 filtration [12]. Liang et al. [13] stated that electrospun pullulan nanofibers with high amylose starch (HAMS) improved thymol loading capacity from 2.38 to 6.15%, demonstrating enhanced antimicrobial properties and sustained release for potential applications in food packaging. Given their widespread use, it is vital to assess the degradation behavior and potential cytotoxicity of nanofibers, as both properties are influenced by environmental conditions, and material composition. Subsequently, comprehensive safety assessments are an essential component in this review in order to highlight the safe applications of electrospun nanofibers.

### Immobilised enzymes electrospun nanofibers: encapsulated and entrapped enzymes

Electrospun nanofibers serve as an excellent support system for enzyme immobilization due to its desirable characteristics including high specific surface area, porosity, ease of use and controllable fiber diameters [14]. While enzymes are valued for their substrate specificity and catalytic efficiency, free enzymes often suffer from structural instability, limiting their activity under harsh conditions [15, 16]. Enzyme immobilization methods, such as encapsulation or entrapment, adsorption, crosslinking and covalent attachment [17], have proven to be an effective method to address these limitations. Immobilization techniques enhance enzyme performance, improve reusability, and facilitate easy separation and operation [18, 19]. Among these techniques, encapsulation and entrapment using electrospun nanofibers have shown promising results, warranting closer examination.

Enzyme encapsulation and entrapment electrospun nanofibers offer superior efficiency, enhanced stability, and easy reusability compared to adsorption or covalent methods. According to Zdarta et al. [20], encapsulation achieved 100% immobilization of laccase in poly (methyl methacrylate)/Iron (II, III) oxide (PMMA/Fe₃O₄) nanofibers, whereas covalent bonding resulted in 79% enzyme immobilization. Furthermore, the encapsulated laccase in PMMA/Fe₃O₄ nanofibers retained 90% of its activity after 40 days, whereas covalently bonded laccase retained only 75%. These findings highlight the potential advantages of encapsulation and entrapment strategies. However, further research is needed to develop systems that combine high enzymatic activity, stability, reusability, and scalability for practical applications. Given these advantages and ongoing challenges, a comprehensive discussion of encapsulation and entrapment techniques within electrospun nanofibers is essential to guide future research and innovation in this rapidly evolving field.

### Electrospinning methods, parameters, and applications for enzymes encapsulation and entrapment electrospun nanofibers

The electrospinning methods used for enzyme encapsulation and entrapment play a crucial role in determining the structural and functional properties of the resulting nanofibers. Several parameters significantly influence the morphology and performance of these nanofibers, such as the spinning solution, processing conditions, and environmental conditions. Encapsulated and entrapped enzymes electrospun nanofibers are being explored intensively as promising support materials for biocatalysis, drug delivery, biosensing, and wastewater treatment [21]. For instance, encapsulated horseradish peroxidase (HRP) electrospun nanofibers (sodium alginate/poly (vinyl chloride)) (SA/PVC) was shown to degraded over 80% of sulfamethoxazole and carbamazepine in wastewater within 24 h [22]. Hence, considering the growing significance of these applications, it is crucial to thoroughly review records of previous studies regarding electrospinning techniques and their influencing parameters to effectively guide the development of high-performance enzyme-loaded nanofibers.

### Research novelty and review of relevant literature

Previous studies have primarily focused on feasibility of electrospun nanofibers with different immobilization methods for various applications, rather than systematically comparing immobilization strategies or optimizing electrospinning conditions. This review aims to fill this gap by providing a comparative evaluation of four immobilization methods and highlighting the optimization of electrospinning parameters in the most effective approach in order to improve enzyme performance. The review begins with an introduction of nanofibers and the fundamental principles of electrospinning. It includes a brief discussion on the degradation and potential cytotoxicity of electrospun nanofibers. The role of electrospun nanofibers as support systems for enzyme immobilization is then explored, followed by an overview of commonly used immobilization techniques. A comparative analysis of these methods is presented, emphasizing their respective advantages and limitations. Subsequently, the effect of electrospinning methods and parameters on the properties of enzyme-loaded nanofibers is examined. The review also explores the applications of encapsulated and entrapped enzymes electrospun nanofibers across various fields. Finally, current challenges and future research directions in the rapidly evolving field of functionalized nanofibers are briefly discussed. Overall, this review aims to summarize key strategies and applications of enzyme entrapment and encapsulation in electropun nanofibers with the goal of inspiring further exploration and development of high-performance systems in this field.

## Nanofibers and basic principles of electrospinning

**Fig. 1:**
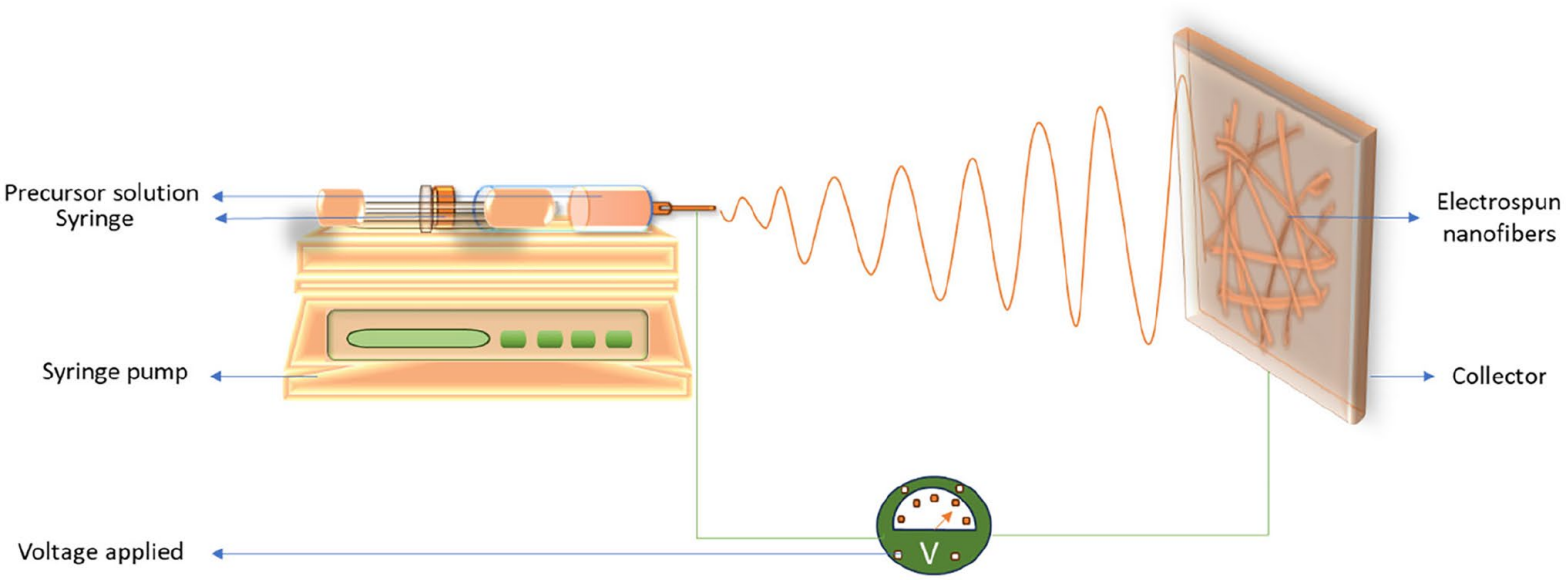
Schematic diagram of conventional electrospinning

In recent years, nanofibers have attracted considerable interest and have been extensively applied across diverse fields including biotechnology, environmental science, and material engineering. These nanostructures can be synthesized from a variety of materials such as polymers, ceramics, metals, or composites. Among these, polymer-based nanofibers are the most widely utilized due to their ease of fabrication, cost-effectiveness, and flexibility [23]. Among the various synthesizing technique, electrospinning has emerged as one of the most versatile and widely adopted technique for nanofibers synthesis. A standard electrospinning setup consists of four main components: a syringe pump, a metallic needle attached to a syringe, a high-voltage power supply, and a conductive collector that is typically made of copper or aluminium (Fig. 1).

The electrospinning process begins when high voltage electric charges are transferred to the polymer, inducing instability within the solution. As the syringe pump drives the polymer solution through the metallic needle towards the collector, the electrostatic forces generated by the applied voltage causes the molecules within solution to repel each other, thus counteracting the surface tension of the droplet at the needle tip. In the absence of a sufficiently strong electric field, the polymer droplet remains spherical due to surface tension. However, as the electric field strength increases, the spherical droplet deforms into a conical structure known as the Taylor cone [24]. The formation of the Taylor cone occurs when the surface tension is balanced by electrostatic forces, resulting in an increased accumulation of charges at the cone’s surface [25]. Once this balance is disrupted by the continued application of voltage, a charged jet of polymer solution is ejected from the apex of the cone and deposited onto the collector. To ensure the stability and continuity of the electrospun jet, the polymer solution must possess adequate cohesive forces to sustain the elongation, stretching, and thinning of the jet as it travels through the electric field [24, 25]. As the jet accelerates toward the collector, the interplay between internal cohesive and external electrostatic forces induces a whipping effect. This dynamic whipping motion further elongates the jet, promoting the formation of nanofibers [26]. As a result, the capability of electrospinning to produce ultrafine, continuous electrospun nanofibers has made it a cornerstone technology in the fabrication of functional nanomaterials for advanced applications.

## Degradation and toxicity aspect of electrospun nanofibers

The degradation behavior of electrospun nanofibers is a critical factor influencing their safety, efficacy, and potential adverse effects, making the assessment of the degradation pathways of both the intermediates and final products essential to ensure their biocompatibility and long-term performance. Accurate evaluation of these degradation processes requires the selection of suitable analytical methods capable of characterizing degradation pathways such as hydrolysis, enzymatic degradation, thermal degradation, pH-dependent corrosion, oxidative decay, bulk and surface erosion, and photodegradation. Each pathway can significantly influence the structural integrity and functional properties of the electrospun nanofibers over time. Moreover, variations in the physicochemical characteristics of nanofibers such as size, shape, surface charge, solubility, and morphology, can further influence not only the nanomaterial’s effectiveness but also their toxicity potential. As such, toxicity assessment must be comprehensive and multidimensional, encompassing in vitro, ex vivo, in vivo, and in silico approaches. Common in vitro assays used to evaluate cytotoxicity include cytocompatibility, trypan blue, comet, apoptosis, 2,7-dichlorofluorescein diacetate (DCFDA), lactate dehydrogenase (LDH) release, live–dead staining, and hemocompatibility assays [27]. More specialized assays such as activated partial thromboplastin time (aPTT) and prothrombin time (PT) for evaluating coagulation, protein adsorption assays, and platelet adhesion tests, are employed to further assess the interaction of functionalized nanofibers with biological systems [28]. For instance, thermogravimetric analysis of clopidogrel-eluting electrospun polyurethane (PU)/polyethylene glycol thromboresistant nanofibrous scaffolds provides valuable insights into both material stability and potential implications for hemocompatibility and toxicity [29]. Among the emerging strategy to address environmental safety concerns is the incorporation of enzymes into the electrospun nanofibers. This approach enables self-triggered and controlled biodegradation, allowing for complete breakdown within a defined period (i.e.: 28 days) while preserving the functional properties of the nanofibers. Additionally, this method has demonstrated minimal toxicity, offering a sustainable and biocompatible approach to mitigate environmental pollution [30]. This understanding of degradation and toxicity is particularly relevant for the design of electrospun nanofiber support systems for enzyme immobilization. The stability of the carrier’s matrix directly affects enzyme retention, activity, and release profiles. Simultaneously, the biocompatibility of the nanofibers ensures minimal adverse interactions with the bioactive compounds. Thus, integrating degradation and toxicity profiles into the material selection and design of electrospun nanofibers provides a solid foundation for the development of safe and efficient immobilized enzyme systems for applications in biocatalysis, biosensing, and biomedical engineering.

## Electrospun nanofibers as support systems for enzyme immobilization

Electrospun nanofibers have emerged as promising support systems for enzyme immobilization due to their accessibility, versatility and broad material compatibility [31]. Each feature allows for enhanced enzyme loading, improved mass transfer, and better retention of enzymatic activity. In general, the enzymes themselves are highly selective biological catalysts and are valued for their environmental friendliness, resulting in the continued expansion of their implementation in industries. In line with this trend, the global enzyme market keeps increasing and has been valued at USD 60.48 billion in 2023. It is projected to grow at a compound annual growth rate (CAGR) of 4.9% between 2024 and 2030 [32]. Notably, the laccase market was estimated at USD 3 million in 2021, and is expected to reach USD 4.54 million by 2031, growing at a CAGR of 4.3% [33]. Despite their many advantages, free enzymes have certain limitations such as low stability, susceptibility to denaturation, and reduced activity under extreme pH, temperature, or solvent conditions. These drawbacks restrict their practical applicability in harsh industrial settings. Enzyme immobilization has proven to be an indispensable method to address these limitations. However, understanding the conformational changes enzymes undergo during the electrospinning process is crucial to maintaining their functionality post-immobilization.

### Commonly utilized enzymes, and the conformational changes during electrospinning

Enzymes are composed of amino acid chains folded into specific three-dimensional structures that determine their catalytic properties. While the primary structure, formed by stable peptide bonds, is generally resistant to disruption, the secondary structures such as *α*-helices and *β*-sheets, which are stabilized by hydrogen bonding, are more susceptible to environmental changes. Enzyme stability is a critical factor in industrial applications as many applications involve extreme conditions that can cause deactivation [34, 35]. Immobilization of enzymes on electrospun nanofibers has demonstrated the ability to mitigate such instability. It is an acknowledged fact that during electrospinning, exposure to solvents, high voltage, and shear stress may induce subtle structural changes in enzymes, such as minor alterations in the *α*-helix to *β*-sheet ratio. Similarly, the tertiary structure, maintained through hydrophobic interactions, ionic bonds, and disulfide bridges, may also undergo slight rearrangements, particularly in surface-exposed regions that are more sensitive to external stimuli. However, these conformational changes are generally limited in scope, and do not lead to complete denaturation or loss of enzymatic function. For instance, immobilization of carbonic anhydrase on electrospun agar–polyacrylonitrile (PAN) nanofibers crosslinked with glutaraldehyde demonstrated improved thermal and pH stability as it retained 83.9% of its catalytic activity and exhibited reusability up to nine cycles [36]. These findings suggest that electrospinning-induced conformational changes were minimal, allowing the enzyme to maintain its functional integrity while gaining enhanced robustness suitable for industrial applications. Similarly, laccase immobilized on cellulose acetate (CA)/chitosan (CS)/poly(ethylene oxide) (PEO) nanofibers underwent only slight conformational changes, as evidenced by Fourier Transform Infrared Spectroscopy (FTIR) spectral shifts at 1560 and 1640 cm⁻^1^, indicative of stable enzyme binding [37]. Overall, these modifications contributed to increased enzyme stability without significantly affecting catalytic performance. The extent of the enzyme’s conformational shifts during electrospinning depends on several factors: intrinsic structural flexibility, the polymer matrix, the solvent, and the processing parameters. Understanding these interactions for each type of enzyme utilized is essential for designing effective and stable immobilized enzyme systems.

#### Oxidoreductases

Oxidoreductases, representing a broad class of enzymes that catalyze oxidation–reduction reactions, play vital roles in biocatalysis, biosensing, environmental remediation, and diagnostics. In recent years, among the most studied oxidoreductases in nanofiber immobilization for industrial and biotechnological processes are laccase, horseradish peroxidase (HRP), and glucose oxidase (GOx) [38, 39].

Laccase, a multi-copper oxidase enzyme naturally produced by bacteria, fungi, and plants, is well-suited for degrading phenolic pollutants and dyes, as well as in biosensor development due to their high catalytic efficiency [40, 41]. Electrospun nanofiber membranes can influence the spatial arrangement and conformational integrity of laccase, affecting steric hindrance and local micro-environmental factors (i.e.: pH and polarity) which in turn impact laccase activity. Lin et al. [42] have stated that laccase can be evenly distributed across electrospun nanofibers without significant changes to overall fiber morphology, providing a stable micro-environment that preserves the structural integrity and conformation of the enzyme’s active site. The immobilized laccase retained its catalytic activity, as reflected by coloration activation energies ranging from 50.89 to 33.62 kJ/mol. It was successfully applied in time–temperature indicators to monitor lactic acid bacteria growth in milk, maintaining consistent performance under fluctuating temperature (4–25 °C) [43].

Horseradish peroxidase (HRP) is a widely studied enzyme which participates in the hydroxylation of various compounds. Its substrate specificity enables the effective degradation of phenolic compounds such as pyrogallol, guaiacol, catechin, and catechol [44]. For example, HRP was covalently immobilized onto sodium hypochlorite-functionalized polyurethane (PU) nanofibers. The resulting HRP–PU system demonstrated increase reusability by retaining 11–33% of its catalytic activity after 4–8 h of operation, and successfully catalyzed the decolorization of Orange II dye [45]. In another study, gelatin electrospun nanofibers crosslinked via HRP enabled plasmid DNA immobilization. The preserved catalytic activity of HRP enabled effective immobilization of lipofectamine/pDNA complexes, sustaining gene transfection and genome editing in cultured cells [46]. Past studies have also immobilized HRP on cellulose acetate–polyamidoamine (CA–PAMAM) nanofibers for the development of a non-invasive colorimetric biosensor for hydrogen peroxide (H₂O₂) detection. In the study, the immobilized enzyme maintained its catalytic activity and sensitivity over a wide concentration, providing accurate measurements over a linear range of 5–500 µM with a detection limit of 1.1 µM [47]. The results demonstrate that CA-PAMAM/HRP nanofibers preserved its structural integrity and enzymatic function during electrospinning, offering a stable and effective platform for non-invasive biosensing applications [47].

Glucose oxidase (GO_x_) is an oxidoreductase that catalyzes the oxidation of *β*-D-glucose to glucono-*δ*-lactone, producing hydrogen peroxide as a byproduct. Its high specificity, stability, and ease of production from *Aspergillus niger* and *Penicillium* species, have enabled applications in biosensing, biocatalysis, food processing, and biofuel cells. GO_x_ immobilized within a polyvinyl alcohol/chitosan/tea (PVA/CS/tea) extract electrospun nanofibrous membrane exhibited over 68% activity retention and 73% deoxidization, effectively reducing oxygen levels to ≤1%, thus inhibiting microbial growth [48]. When immobilized onto polyaniline (PANI) nanofibers, GO_x_ exhibited enhanced enzyme activity suitable for biofuel cells application [49]. Similarly, UV-irradiated polyvinyl alcohol/polyacrylonitrile (PVA/PAN) electrospun nanofibers improved GO_x_ enzyme binding, resulting in a 33% increase in catalytic activity, preserved the structural integrity, and stable performance over 12 cycles [50]. Collectively, these studies demonstrate that electrospun nanofibers can effectively maintain the structural integrity and catalytic function of oxidoreductases post-immobilization, highlighting their potential as versatile platforms for food processing, pharmaceuticals, and environmental biocatalysis.

#### Hydrolyses

Hydrolases constitute a class of enzymes that catalyze the cleavage of chemical bonds via hydrolysis. These enzymes facilitate the breakdown of a wide range of substrates in various biological and industrial processes including food processing, pharmaceuticals, and biofuel production. Within this diverse group, enzymes are further categorized into different subclasses based on the specific bonds they target for cleavage. Key subclasses of hydrolases such as lipases, cellulases, lactases, lysozymes, and *β*-galactosidases, are increasingly utilized in biocatalytic applications, particularly when immobilized on nanofibers to enhance their stability and efficiency.

Lipases are multifunctional enzymes that primarily catalyze the hydrolysis of long-chain triglycerides into glycerol and free fatty acids. Beyond hydrolysis, they also catalyze esterification, transesterification, and interesterification reactions in low-water, or non-aqueous systems [51, 52]. In a past study, the immobilization of lipase on magnetic nanocellulose/polyethersulfone nanofibers resulted in the enhancement of its thermal stability and catalytic efficiency. Structural characterization confirmed successful enzyme attachment, and under optimized conditions, the immobilized lipase retained high catalytic activity over five esterification cycles, with a half-life of 120 h [53]. Another study took the approach of entrapping lipase in polylactic acid (PLA) and polyvinylpyrrolidone (PVP) electrospun nanofibers, forming the lipase–PLA and lipase–PVP systems. Entrapment of lipase in PLA and PVP nanofibers yielded biocatalytic activities ranging from 28 to 290 U/g for lipase–PLA, and 182 to 453 U/g for lipase–PVP [54]. A recombinant *Psychrobacter* sp. C18 lipase immobilized on bilayer polyvinyl alcohol/chitosan/zinc oxide (PVA/CS/ZnO) and polycaprolactone/chitosan (PCL/CS) nanofibers showed enhanced pH and thermal stability, storage durability, and tolerance to metal ions [55].

Cellulases, another important subclass of hydrolases, are widely applied in food, pharmaceuticals, textiles, and biorefinery processes. These complex enzymes work synergistically to hydrolyse cellulose and have also been used in enzyme-responsive delivery systems for controlled release applications [56]. Likewies, *β*-galactosidase, when immobilized on polystyrene/functionalized graphene oxide-3-aminopropyl triethoxysilane (PS/GO-APTES) nanofibers, achieved an immobilization efficiency of 87%, 72% efficiency in galacto-oligosaccharide (GOS) synthesis and 81% lactose conversion relative to the free enzyme [57]. Another formulation where *β*-galactosidase was encapsulated within PVA nanofibers achieved siamenoside I production rates of 118 ± 0.08 mg/L·h·g. [58]. Additionally, lactase immobilized on polycaprolactone/silk fibroin (PCL/SF) nanofibers demonstrated effective hydrolysis of lactose in milk, achieving 42% efficiency for cow’s milk and 21% for goat’s milk, while showing no toxicity in zebrafish embryo assays [59, 60]. Moreover, lactase–PCL/SF lysozyme, encapsulated in PVA nanofibers, is capable of achieving a drug loading content of 50%, indicating its potential for combined antimicrobial and catalytic applications [61]. Collectively, these findings underscore the ability of electrospun nanofibers in supporting the immobilization of hydrolase enzymes. Their ability to retain enzymatic activity, stability, and reusability makes them valuable platforms for practical applications.

### Immobilized enzyme electrospun nanofibers

**Fig. 2:**
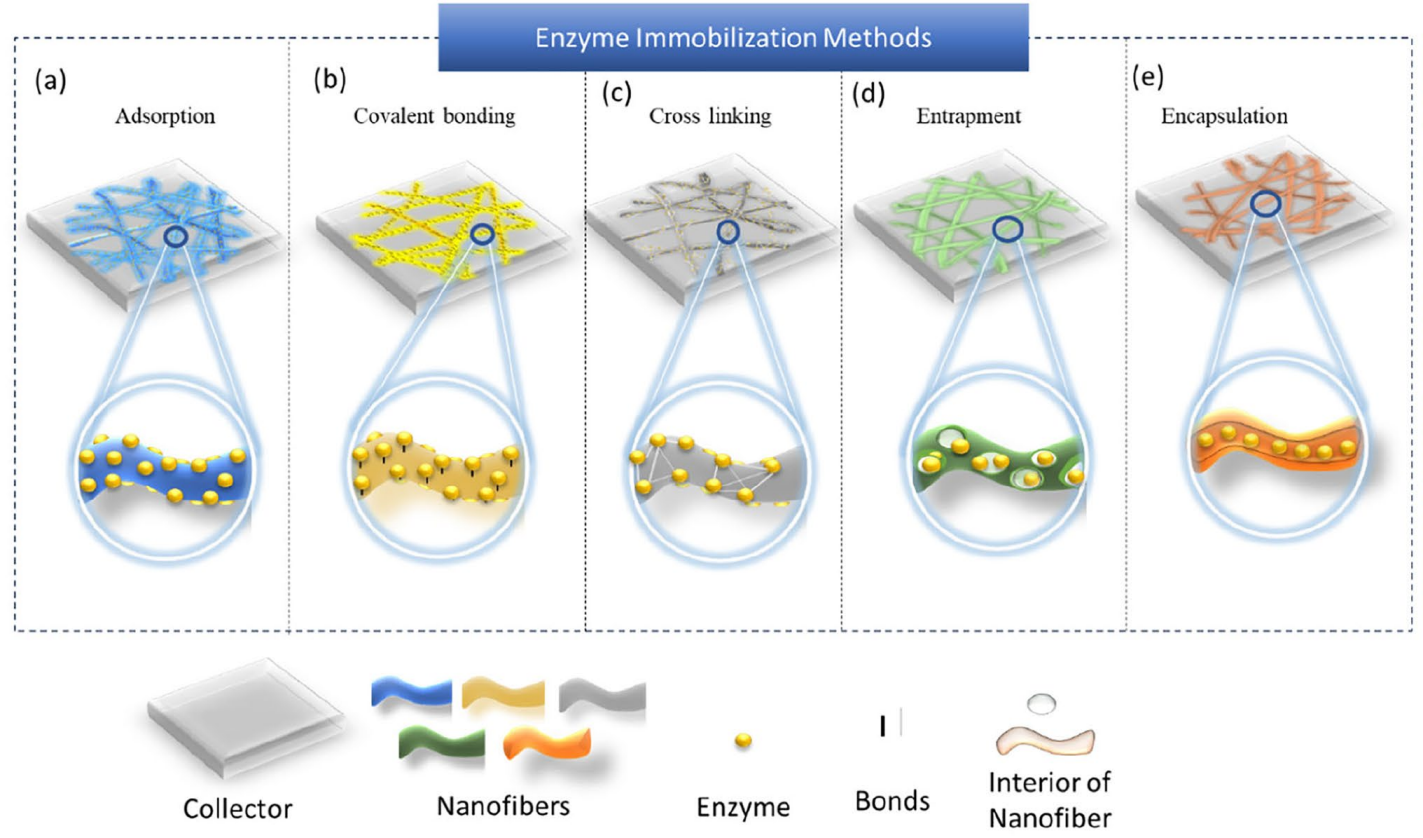
(**a**) Enzyme adsorbed onto the support; (**b**) Enzyme covalently bonded to the support; (**c**) Enzyme crosslinked on the support; (**d**) Enzyme entrapped within nanofibers; (**e**) Enzyme encapsulated in nanofibers

Immobilization of enzymes within electrospun nanofibers enhances enzymatic performance by improving enzyme activity, prolonging storage stability, increasing reusability, and tolerance to extreme operational conditions as high temperatures and pH variations. Electrospun nanofibers can be employed effectively as supports for enzyme immobilization via various methods, including adsorption, covalent binding, crosslinking, entrapment and encapsulation (Fig. 2).

The selection of immobilization method depends on multiple factors: the chemical composition and properties of the nanofiber support, the type of enzyme or biomolecule, and the conditions of the catalytic reaction. A strategic alignment between the enzyme and the nanofiber matrix is critical to preserve the enzyme’s functional conformation and maximize its catalytic activity. In particular, parameters such as surface area, porosity, and biocompatibility of the nanofiber scaffolds are essential in determining the efficiency of enzyme loading, accessibility of active sites, and retention of enzymatic activity. The versatility and adaptability of these nanofiber features make them a compelling option for immobilized enzyme applications. The following subsections detail the primary methods of enzyme immobilization on electrospun nanofibers, highlighting their respective mechanisms, advantages, limitations, and application-specific outcomes.

#### Adsorption of enzyme onto nanofibers

Adsorption is a widely utilized method of enzyme immobilization that involves attaching enzymes to the surface of a support material through physical interactions. The main interactions between enzymes and support are ionic interactions, hydrogen bonds, and van der Waals forces. The simplicity of this technique makes it especially attractive for sensitive enzymes. Recent studies have demonstrated the effectiveness of adsorbed enzyme electrospun nanofibers across various applications. For instance, lipase immobilized on recycled poly (ethylene terephthalate)/cellulose acetate (PET/CA) nanofibers via adsorption retained 50% activity after 13 cycles and 70% after 21 days, exhibiting enhanced stability for wastewater treatment despite lower enzymatic activity (21.96 U) than free lipase [62]. A study by Mercante et al. [63] also stated that adsorbed GO_x_-electrospun nanofibres achieved glucose detection limits as low as 14 μM, making it suitable for bioelectronic glucose monitoring. GO_x_-polyurethane/CS-PANI-ZnO and GO_x_-silicon carbide nanofibrous biosensors demonstrated high glucose sensitivity with detection ranges of 0.01–9.48 mM and 0.5–20 mM, respectively [64, 65]. *β*-galactosidase immobilized on PS/GO-APTES retained 80% of its activity over 60 days and 54% after 20 cycles, showcasing its applicability in galacto-oligosaccharide (GOS) synthesis and lactose conversion in food biocatalysis [66]. Additionally, carbon monoxide dehydrogenase immobilized on chitosan/copper (II) benzene-1,3,5-tricarboxylate electrospun nanofiber exhibited outstanding carbon monoxide sensitivity (1.76%/ppm), with a detection limit of ~0.85 ppm, and rapid response/recovery times of 10/20 s [67]. Meanwhile, laccase adsorbed onto polyurethane/regenerated cellulose (PU/RC) electrospun nanofibers membranes via metal ion coordination exhibited high enzyme loading efficiency (up to 136 mg/g) and excellent stability, retaining over 80% of its activity after 20 days. The immobilized enzyme achieved an 81.49% degradation efficiency of 10 mg/L P-chlorophenol and maintained over 58% degradation after multiple reuse cycles [42]. Objectively, while adsorption is a widely used method for immobilizing laccase, it may induce conformational alterations which may negatively influence the enzymatic activity [68]. Nonetheless, these studies highlighted the capability of adsorption to remains relevant, owing to its simplicity, lack of pore diffusion limitations, and suitability for large-scale applications.

#### Covalent bonding of enzyme onto nanofibers

Covalent binding involves the formation of stable chemical linkages between functional groups on the enzyme and reactive groups on the nanofiber surface. It provides strong and stable bonding, significantly minimizing enzyme leaching and enhancing structural stability. However, covalent binding can sometimes involve active site residues, which may alter the conformation of enzymes and reduce their catalytic activity. Despite this, the reduced conformational flexibility and thermal motion generally contribute to improved enzyme stability, making covalent binding a favourable immobilization strategy. Recent studies have reported improvements in enzyme performance using covalent bonding in nanofibrous systems, leading to enhanced stability, enzymatic activity, and reusability.

A past study involving *β*-glucuronidase exhibited a 3.4-fold increase in hydrolytic activity, while laccase retained high activity and pollutant degradation efficiency after immobilization [69]. Covalent immobilization has also enabled highly sensitive biosensors, achieving detection limits of 4.7 nM for *β*-glucuronidase, 5 µM for acetylcholine, and 1.045 µM for monosodium glutamate [70]. Biocatalytic applications benefited from immobilized laccase, which retained over 60% reusability after multiple cycles, effectively degrading environmental pollutants [71]. Furthermore, in biomedical applications, apyrase and 5'-nucleotidase nanofibers significantly reduced platelet aggregation, supporting their use in antithrombosis and endothelialisation [72]. According to El-Aassar [73], laccase immobilized on poly(acrylonitrile-co-styrene)/pyrrole via covalent binding achieved a degradation efficiency of 74%. These findings highlight covalent bonding as a reliable strategy for enzyme immobilization, ensuring high activity retention, stability, and reusability for applications in biocatalysis, biosensing, and biomedical engineering.

#### Crosslinking of enzyme onto nanofibers

The key distinction between the crosslinking and covalent binding methods lies in how the enzymes are attached. In the crosslinking method, enzymes are covalently linked to each other using a multifunctional reagent, whereas in the covalent binding method, enzymes are directly bound to the support via covalent bonds. Additionally, these crosslinked enzyme aggregates (CLEAs) are then anchored to the nanofiber surface, stabilizing their structure through both intermolecular connections and attachment to the support material [21, 31]. Glutaraldehyde is the most commonly used crosslinking agent for enzyme immobilization on electrospun fibers [74, 75]. Chemical crosslinking can improve the structural integrity and durability of the enzyme-nanofiber systems [76]. These studies highlight the effectiveness of crosslinking in stabilizing immobilized enzyme electrospun nanofibers biocatalysts. According to Hong et al. [77], PVA/Nylon 6/CS-based nanofibers retained 75–84% of their enzymatic activity after 30 days at 4 °C, outperforming free enzymes that retained only 48–56%. Reusability was also improved, with 35–42% activity retained over ≥6 cycles, demonstrating prolonged catalytic efficiency. Moreover, Hsieh et al. [78] stated that despite slight increases in *K*_m_ values (from 200 to 268 μM), the immobilized enzymes exhibited high stability and activity retention at elevated temperatures (50–60 °C, pH 8.5). These findings reinforce crosslinking as a promising method for producing robust, reusable enzyme-based biocatalysts.

#### Entrapment of enzyme in nanofibers

Entrapment involves entrapping the enzyme within the porous structure of the nanofibers, allowing free diffusion of the substrates while restricting enzyme leaching [79]. Unlike encapsulation, where enzymes are enclosed within a fiber’s core and may result in conformation alteration or partially blocking of the active sites, entrapment distributes enzymes throughout the nanofiber matrix, preserving their native structure and catalytic accessibility (Fig. 2). The pore size and structure of the support material are critical to optimizing entrapment. Diffusional constraints can hinder catalytic performance when substrates have high molecular weights. If pores are too large, enzyme leakage may occur; if too small, substrate diffusion may be hindered, particularly for large molecules [68, 80, 81]. Designing nanofibers with appropriate porosity and high surface area enhances both enzyme retention and catalytic performance. Additionally, this method often has a high degree of reusability, which is desired in industrial applications [80, 81]. Several studies have demonstrated the advantages of enzyme entrapment in nanofibers structures. For example, GO_x_ entrapped in PVA nanofibers formed highly porous structures with good catalytic properties [82]. In another study, Sakai et al. [83] developed electrospun PVA membranes with lipase entrapped in silicate cages that achieved 4.5-fold higher transesterification activity in isooctane compared to unmodified fibers, demonstrating well-retained enzyme activity. These studies highlight the effectiveness of enzyme entrapment in nanofibers to preserve enzyme structure while enhancing reusability and stability for industrial applications.

#### Encapsulation of enzyme in nanofibers

Encapsulation typically uses a core–shell electrospinning configuration, where the enzyme is fully enclosed within the nanofiber [84]. Encapsulation and entrapment both methods entrap enzymes in the polymer. To develop this nanofiber system, the enzymes are first mixed properly with the polymeric solution until a uniform solution is obtained. The polymeric solution completely wraps the enzymes, followed by electrospinning to produce enzyme encapsulated nanofibers. The enzymes are encapsulated inside the nanofibers as depicted in Fig. 2.

Ishiguro et al. [85] encapsulated lactase in core–shell electrospun nanofibers using a 10 wt% nylon solution in 2,2,2-trifluoroethanol (TFE), a 16.6 wt% poly(acrylamide)-co-poly(diacetone–acrylamide/adipic dihydrazide (poly(AM/DAAM)/ADH) solution, and 1 wt% lactase. The immobilized lactase recorded 114% enzymatic activity and 95% reusability after 10 cycles. The nanofibers exhibited strong mechanical properties, with a maximum load capacity of 1.18 ± 0.13 N and an elongation to failure of 21.3 ± 3.3%. In addition, Hosseini et al. [86] also reported superior stability and reusability of their encapsulated *Bacillus licheniformis* *α*-amylase (BLA) compared to its free form. Under optimal conditions (pH 6.6, 50 °C), immobilized BLA retained over 75% activity after 45 days, while free BLA retained only 30%. It also exhibited improved substrate affinity (*K*_m_: 4.2 ± 0.4 mg/mL vs 5.5 ± 0.3 mg/mL for free BLA), maintaining nearly 100% activity after 10 cycles and 50% after 15 cycles, despite a slightly lower *V*_max_ (1.9 ± 0.2 µmol/mL·min vs 2.1 ± 0.5 µmol/mL·min).

Giraldi et al. [87] encapsulated *Trametes versicolor* laccase (TVL) in poly(L-lactic acid) (PLLA) nanofibers, achieving 54% immobilization efficiency and 18% retained activity. Ojstršek et al. [88] encapsulated *Candida rugosa* lipase (LI) and *Glycine max* lipoxygenase (LOX) in a poly(glycerol) poly(ricinoleate) (PGPR) membrane via emulsion electrospinning. The optimized water-in-oil emulsion contained 2% w/v polyoxyethylene (20) sorbitan monolaurate (Tween20) and 0.1 M sodium chloride (NaCl) in the aqueous phase and 6% PGPR in edible oil as the oil phase at an 80/20 w/w ratio. Electrospinning was performed at 55 kV, a 20 cm needle-to-collector distance, and a 0.54 mL/h flow rate. The resulting encapsulated enzyme nanofibers demonstrated effective emulsion stability, with droplets averaging 391.0 ± 15.6 nm in size, a low polydispersity index (0.255 ± 0.07), and good gravitational stability after 14 days.

The encapsulation of horseradish peroxidase (HRP) in PVC improved enzyme stability and reusability, retaining over 60% enzymatic activity after 20 days at 4 °C, while free HRP had no recycling potential. HRP-PVC maintained >60% activity after 10 cycles, whereas free HRP dropped to <20% over the same period. Kinetic analysis showed a slight decrease in substrate affinity (*K*_m_: 1.8 mM vs. 1.54 mM for free HRP) and a lower reaction rate (*V*_max_: 312 ± 20 U/mg vs. 422 ± 4 U/mg for free HRP), likely due to diffusion limitations. Despite this, HRP-PVC offers enhanced durability, making it a promising approach for pollutant removal [22]. These studies demonstrate that enzyme encapsulation via electrospinning enhances enzyme stability, reusability, and mechanical properties while maintaining significant enzymatic activity, making it a promising strategy for large scale applications.

### Recent advancements, patents and clinical trials of immobilised enzyme electrospun nanofibers

Recent advancements have been made in the development of encapsulated and entrapped enzyme electrospun nanofibers, particularly through techniques such as blend, emulsion, and co-axial electrospinning, with ongoing efforts aimed to improve enzyme distribution within the fiber matrix, minimizing denaturation during processing, and enhancing the overall enzymatic activity. Another emerging trend involves incorporating functional additives into nanofibers to introduce more reactive sites for enzymes [45]. These modifications not only increase mechanical stability and conductivity but also help preserve enzymatic activity under stressful operational conditions. To guide optimization efforts, a variety of structural and functional characterization tools such as Scanning Electron Microscopy (SEM), Fourier Transform Infrared Spectroscopy (FTIR), enzymatic activity assays are being increasingly utilized to correlate physical properties with catalytic performance. According to data from Lens.org, the terms “electrospun” and “nanofiber” yielded over 12,806 patents and 3633 articles. Of these, 1135 patents and 3479 articles are specifically related to enzyme immobilization, reflecting a constantly growing research and commercial interest. Although the electrospinning method was first patented by Cooley in 1900, it gained prominence in the late twentieth century with the rise of nanotechnology. Since then, electrospun nanofibers have found widespread applications in catalysis, drug delivery, tissue engineering, wound healing, antimicrobial therapy, and theranostics, owing to their biodegradability, biocompatibility, and functional tunability [89, 90]. Recent patents have underscored the growing interest in the diverse applications of electrospun nanofibers for enzyme immobilization. For example, WO 2020025793 A1 discloses a beaded electrospun nanofiber membrane for poorly water-soluble agents [91], while CZ 303244 B6 outlines the utilization of nanofiber carriers for active biomolecules [92]. Both findings demonstrate principles adaptable for enzyme immobilization. A patent entitled *Nanofibrous Materials as Drug, Protein*,* or Genetic Release Vehicles* is particularly relevant, as enzymes are a type of protein; therefore, systems designed for protein delivery can be effectively adapted for enzyme immobilization [93]. Patents such as EP 3253424 B1 showcase antimicrobial applications of immobilized enzyme nanofibers [94], while US 9163338 B2 highlights electrospun chitosan nanofiber mats as robust and modifiability supports for immobilized enzymes [95]. Further notable disclosures include KR 20170053288 A (polymer–enzyme electrospinning process) [96], CN 111876405 A (enzyme immobilization on nanofibers) [97], and CN 103300071 A (modified nanofiber composite membranes for enzyme fixation) [98]. These patents collectively emphasize the technological potential and functional adaptability of electrospun nanofibers in enzyme immobilization. They lay the groundwork for innovation across multiple fields, from biosensing to environmental remediation. However, despite these promising developments, it is important to note that none have yet to progress into clinical trials or human-based studies.

## Comparison of enzyme immobilization methods

In the comparison of laccase immobilization methods on Nylon 6, covalent binding via 1-Ethyl-3-(3-dimethylaminopropyl) carbodiimide/N-Hydroxysuccinimide (EDC/NHS) proved to be superior to adsorption in terms of enzyme loading, stability, and reusability [99]. Covalently bonded laccase exhibited higher laccase loading (423 mg/g) compared to adsorbed laccase (282 mg/g). The storage stability of covalently bonded laccase was significantly better, with 95% retention after 30 days, compared to 60% for adsorbed laccase and only 31% for free laccase. In terms of reusability, covalent binding also outperformed adsorption, maintaining 70% activity after 10 cycles, while adsorbed laccase retained 61%. Furthermore, covalent binding showed superior dye removal efficiency, with 77% removal of Reactive Blue 4 and 63% removal of Reactive Black 5, indicating better performance for dye degradation. Overall, covalent binding is the preferred method for long-term applications, offering higher enzyme retention, enhanced stability, and better reusability for processes such as dye removal.

According to Bayazid et al. [100], lysozyme immobilized on bacterial cellulose nanofibers through physical adsorption maintained over 70% of its original activity after nine reuse cycles, demonstrating good storage stability and enhanced antimicrobial effectiveness despite a modest 12% reduction in activity. In contrast, covalent bonding typically provides stronger enzyme attachment but can limit enzyme flexibility and activity. In this study, adsorption offers a simpler preparation method while still ensuring sufficient reusability and performance for practical applications.

Aldea et al. [101] also stated that immobilized GO_x_ on a composite of gold/PMMA/polyethylene terephthalate using different methods: cross-linking with glutaraldehyde, physical adsorption, and covalent binding using 11-mercaptoundodecanoic acid and EDC/NHS. The results showed that covalent immobilization provided higher stability compared to both physical adsorption and cross-linking methods. The GO_x_ biosensor constructed via covalent immobilization exhibited a sensitivity of 3.10 μA/cm^2^. mM and a detection limit of 0.33 mM, making it effective for glucose detection. This suggests that covalent binding offers better long-term stability and more reliable performance than the other immobilization methods tested.

Sass and Jördening [102] also compared the performance of covalently bonded and encapsulated *β*-galactosidase from Aspergillus oryzae using gelatin fibers as supports. The results showed that covalently bonded *β*-galactosidase achieved a GOS yield of 45% and a lactose conversion of 15%. The encapsulated *β*-galactosidase exhibited significantly higher performance, with 72% GOS yield and 31% lactose conversion. The key difference was that encapsulated enzymes retained their activity during the reaction, likely because the enzyme’s active site was not blocked by the substrate, allowing for greater enzyme functionality and efficiency in the conversion process. This suggests that encapsulation may provide better protection and access to the enzyme’s active site compared to covalent bonding.

Alvarado-Ramírez et al. [103] reported that among the adsorption, covalent bonding and entrapment immobilization methods, entrapment demonstrated the best performance, with the highest specific activity (0.1147 U/mg for PVA, 0.0896 U/mg for PVA/SA, and 0.1037 U/mg for PVA/CS) and the highest immobilization rate (95–97%). PVA/SA nanofibers via entrapment method showed the best storage stability (95.18% after 4 weeks) and reusability (77.85% after 5 cycles), whereas PVA and PVA/CS lost most of their activity after multiple cycles. Covalent bonding exhibited moderate specific activity (0.0734–0.0756 U/mg) and immobilization efficiency (56–76%), with PVA/CS showing the highest storage stability (66.52%) and PVA demonstrating the best reusability (63.26% after 5 cycles). However, adsorption was the least effective method, with the lowest specific activity (0.0102–0.0196 U/mg), immobilization rate (53–61%), and poor reusability, with all nanofibers retaining less than 15% activity after 5 cycles. Overall, entrapment, particularly in PVA/SA nanofibers, was the most efficient approach, offering superior enzyme stability and retention. These studies show the potential of encapsulated and entrapped enzyme electrospun nanofibers for further study.

## Electrospinning method for enzymes encapsulation and entrapment

**Fig. 3:**
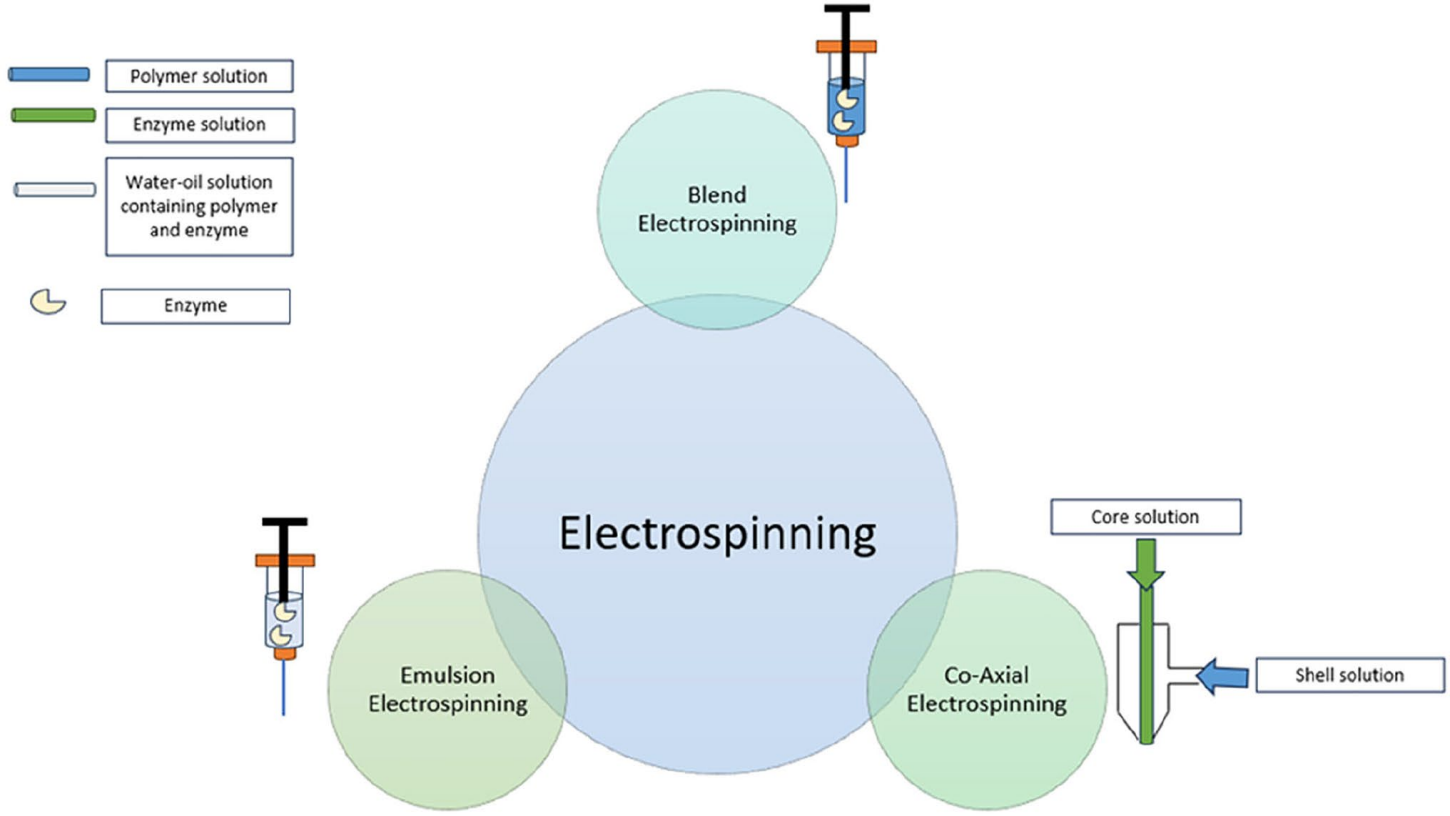
Schematic diagram of electrospinning method

Long operation time of the conventional electrospinning process poses a significant challenge for large-scale applications. With a typical electrospun nanofibers production rate ranging from 0.01 to 1 g/h, this process is inadequate to meet industrial demands [104]. Conventional electrospinning is primarily employed to electrospun polymeric solutions to form nanofibers, rather than loaded enzymes directly in the process. To address these challenges, a lot of research has gone into developing novel electrospinning including coaxial, emulsion and blend electrospinning for enzyme encapsulation and entrapment (Fig. 3).

### Coaxial electrospinning process

The coaxial electrospinning process is similar to the conventional electrospinning process but incorporates a coaxial needle where concentrically aligned, dual syringes are connected to a high-voltage source. In coaxial electrospinning, two fluids are dispensed simultaneously. This method enables the immobilization of enzymes in core–shell electrospun nanofibers. The core solution is pumped through an inner needle and the shell solution through an outer needle. The core–shell structure enables the encapsulation of enzymes that were previously incompatible with conventional electrospinning, forming the core portion of nanofibers while the protective shell shields them from harsh external conditions. Onyekuru et al. [105] reported coaxial electrospinning is a preferable choice for optimizing enzyme retention because it has been demonstrated to achieve higher enzyme loading (21.7%) and encapsulation efficiency (94.6 ± 5.4%) compared to blend electrospinning (19.2% loading and 86.2 ± 2.9% efficiency). Blend electrospinning, on the other hand, preserves enzyme bioactivity while providing a less complicated and straightforward processing technique. These findings showed that the choice between coaxial and blend electrospinning depends on the specific application, balancing between process simplicity and encapsulation efficiency. Ishiguro et al. [85] confirmed the formation of encapsulated enzyme electrospun nanofibers by using this method. PVA/GO_x_ and PVA/glucose dopes were employed to produce fully entangled coaxial electrospun nanofibers with an average diameter of 475.8 nm [106]. These recent studies have demonstrated the feasibility of coaxial electrospinning for enzyme encapsulation.

### Emulsion electrospinning process

Emulsion electrospinning provides advantages over co-axial and conventional electrospinning in terms of scalability [104]. Unlike conventional electrospinning, which requires highly viscous polymer solutions, emulsion electrospinning can produce high-quality nanofibers using low molecular weight and diluted polymer solutions. This method is also effective for creating core–shell nanofiber structures using a single-nozzle setup [9]. A stable emulsion requires three essential components: (a) the oil phase, (b) aqueous phase, and (c) surfactants or emulsifiers. Zhang et al. [107] successfully produced core–sheath PCL/PEG nanofibers encapsulating fluorescently labelled bovine serum albumin via coaxial electrospinning. Table 1 shows recent studies on the encapsulation and entrapment of various enzymes in electrospun nanofibers using emulsion electrospinning.

**Table 1:** Recent studies on solution parameter, ambient parameter and processing parameter of synthesis of entrapped or encapsulated enzyme coaxial electrospun nanofibers

Enzyme-carriers electrospun nanofibers	Immobilization method	Optimal solution parameters	Optimal ambient parameters	Optimal processing parameter	Results	Reference
*Aspergillus oryzae* lactase- poly(acrylamide)-co-poly(diacetone–acrylamide)/adipic dihydrazide-Nylon 6 (poly (AM/DAAM)/ADH-Nylon 6)	Encapsulation	Nylon 6 solution: 10 wt% and in TFE Poly(AM/DAAM)/ ADH solution: 16.6 wt% Poly (AM/DAAM)/ ADH (0.5 molar equivalent with respect to DAAM unit in poly (AM/DAAM) in 100 nM phosphate buffer (PBS) Lactase: 1 wt% with respect to poly (AM/DAAM) mass)	–	Voltage: 20 kV Distance: 15 cm Flow rate: 0.2 mL/h 1 mL/h 1.2 mL/h	Homogeneous stacked structure Average diameter: 420 ± 115 nm Enzymatic activity: 114% Reusability: 95% after 10 cycles Maximum load: 1.18 ± 0.13N Elongation to failure: 21.3 ± 3.3	Ishiguro et al. (2022) [85]
Alkaline phosphatase-poly(ethylene oxide) (ALP-PEO)	Encapsulation	PEO solution: 600 kDa 3% w/v and in ethanol: water (7:3 v/v) ALP solution: 5% w/v and in PBS	Temperature: 25 ± 2 °C Relative humidity: 30 ± 1%	Voltage: 12 kV Distance: 15 cm Flow rate: 0.1 mL/h 0.6 mL/h	Bead-free cylindrical structure Average diameter: 316 ± 127 nm	Onyekuru et al. (2021) [105]
Glucose oxidase/polyvinyl alcohol-glucose/polyvinyl alcohol (GO_x_/PVA-glucose/PVA)	Encapsulation	PVA/GO_x_ (4:1): 4 mL PVA/1 mL 2% GO_x_ in PBS PVA/glucose (4:1): 4 mL PVA/1 mL glucose in PBS	-	Voltage: 15.5 kV to 18.5 kV Distance: 11 cm Flow rate: 0.54 mL/h	Fully entangled and straight thinner structure (GOx/PVA–PVA glucose) Average diameter: 475.8 nm	Leonarta, & Lee (2021) [106]

### Blend electrospinning process

Blend electrospinning, also known as co-electrospinning using a single syringe, involves mixing two materials and electrospinning them into nanofibers in a single step. This method is simple, widely used, and often does not require additional chemical or thermal treatment. It allows for high enzyme loading and uniform dispersion of biomolecules. The enzymatic tertiary structure appears to be unaffected by the high voltage used during the procedure. However, one drawback is the potential loss of enzyme activity due to exposure to solvents or the electric field. Additionally, using a single syringe to process heterogeneous mixtures can increase the risk of nozzle clogging [108]. Recent studies have focused on the encapsulated and entrapment enzymes using blend electrospinning compared to other methods such as coaxial electrospinning, coaxial electrospinning and emulsion electrospinning as summarized in Tables 1, 2, and 3.

**Table 2:** Recent studies on solution parameter, ambient parameter and processing parameter of synthesis of entrapped or encapsulated enzyme emulsion electrospun nanofibers

Enzyme-carriers electrospun nanofibers	Immobilization method	Optimal solution parameters	Optimal ambient parameters	Optimal processing parameter	Results	Reference
*Candida rugosa* lipase (LI)/ *Glycine max* lipoxygenase (LOX)/polyoxyethylene (20) sorbitan monolaurate (Tween20)/ sodium chloride (NaCl)- poly (glycerol) poly(ricinoleate) (PGPR)	Encapsulation	Tween20-NaCl/PGPR (80/20w/w): 2% w/v Tween20 and 0.1 M NaCl in water/6% PGPR in edible oil) LI/LOX: 1:1 ratio PEO: 5%w/v	Temperature: 20 ± 5 °C Relative humidity: 51%	Voltage: 55 kV Distance: 20 cm Flow rate: 0.54 mL/h	Average droplet size: 391.0 ± 15.6 nm Polydispersity index: 0.255 ± 0.07 Gravitational stability: Good after 14 days	Ojstršek et al. (2024) [88]
*Trametes versicolor* laccase-poly(L-lactic acid) (TVL-PLLA)	Encapsulation	PLLA pellets: 300 mg and in dichloromethane (DCM)/ N,N-dimethylformamide (DMF) (92.68/7.32 wt.%) Pluronic©F127: 33.4 mg TVL solution (3.2 and 5.1% w/w): 10 and 16 mg TVL in 0.20 mL of Milli-Q water	-	Voltage: 17 kV Distance: 20 cm Flowrate: 1.38 mL/h	Regular bead-free Average diameter: 810 ± 290 nm (TVL-5.1% w/w PLLA) and 420 ± 160 nm (PLLA-Pluronic) For TVL-3.2%w/w PLLA, Immobilization efficiency: 54% Retained activity: 18% Enzymatic activity: 0.0047 ± 0.00064 U/mg mat Specific activity: 0.27 ± 0.037 U/mg	Giraldi et al. (2022) [87]
*Bacillus licheniformis* α-amylase-ethyl cellulose (BLA-EC) with emulsion loaded	Encapsulation	EC solution: 20%w/v in acetic acid/ethyl acetate (20:80 v/v) 2%w/v BLA and 2%w/v poly ethylene glycol (PEG) Viscosity: 697 ± 23 mPa·s Conductivity: 27 ± 5 μS/cm Surface tension: 59 mN/m	Temperature: 25 ± 2 °C Relative humidity: 30 ± 1%	Voltage: 18 kV Distance: 10 cm Flowrate: 1 mL/h	Uniform structure Relative activity: 90% The enzyme loading efficiency: 5 mg/g Spinnability: + + + + Optimal condition: pH 6.6 and 50 °C (immobilized BLA) and pH 6.6 and 50 °C (free BLA) Storage stability: >75% after 45 days (immobilized BLA) and 30% after 45 days (free BLA) Reusability: ~100% and 50% after 10 and 15 cycles (immobilized BLA) *K*_m_: 4.2 ± 0.4b mg/mL (immobilized BLA) and 5.5 ± 0.3a mg/mL (free BLA) *V*_max_: 1.9 ± 0.2 µmol/mL·min (immobilized BLA) and 2.1 ± 0.5 µmol/mL·min (free BLA) Relative activity: 90% (immobilized BLA)	Hosseini et al. (2022) [86]
*Petroselinum crispum* phenylalanine ammonia lyase (PcPAL)- polylactic acid (PLA)	Entrapment	PcPAL solution (0.15 w/w%): 1.79 mg/mL and in Tris (hydroxymethyl)aminomethane (Tris) buffer PLA solution (8% w/w): 1 g and in DCM-DMF (6:1 v/v)	Temperature: 25 °C	Voltage: 10–20 kV Distance: 10–15 cm Flowrate: 0.48–6 mL/h	Uniform structure Average diameter: 457 ± 84 nm Specific enzyme loading: 30 U/g Viscosity: 561 ± 92 mPas	Koplányi et al. (2021) [111]

**Table 3:** Recent studies on solution parameter, ambient parameter and processing parameter of synthesis of entrapped or encapsulated enzyme blend electrospun nanofibers

Enzyme-carriers electrospun nanofibers	Immobilization method	Optimal solution parameters	Optimal ambient parameters	Optimal processing parameter	Results	Reference
Alkaline phosphatase-poly(ethylene oxide) (ALP-PEO)	Encapsulation	PEO solution: 600 kDa, 3% w/v and in ethanol:water (7:3 v/v) ALP solution: 5% w/v and in PBS	Temperature: 25 ± 2 °C Relative humidity: 30 ± 1%	Voltage: 22.5 kV Distance: 10 cm Flow rate: 0.8 mL/h	Smooth and uniform bead-free structure Average diameter: 236 ± 79 nm	Onyekuru et al. (2021) [105]
*Vigna unguiculata* phytase-polyvinyl alcohol (VuPhy-PVA)	Entrapment	PVA (8% w/v) in 10 mL 0.1 M sodium acetate (NaOAc) buffer 1.5% w/v VuPhy	Temperature: 25 °C	Voltage: 23 kV Distance: 14 cm Flowrate: 0.5 mL/h	Average diameter: 35.79 ± 15.24 nm	Duru Kamaci, & Peksel (2021) [112]
*Vigna unguiculata* phytase-polyvinyl alcohol/sodium alginate (VuPhy-PVA/SA)	Entrapment	SA (2%, w/v) and PVA (10% w/v) in 10 mL 0.1 M NaOAc buffer at 80:20 (v: v) ratio 1.5% w/v VuPhy	Temperature: 25 °C	Voltage: 23 kV Distance: 14 cm Flowrate: 0.3 mL/h	Average diameter: 32.2 ± 18.2 nm	Duru Kamaci, & Peksel (2021) [113]
*Burkholderia cepacia* lipase-polyvinyl alcohol (BcL-PVA)	Entrapment	BcL solution: 100 mg/mL and 50 mM PBS	-	Voltage: 23 kV Distance: 14 cm Flowrate: 0.5 mL/h	Specific enzymatic: 90.6 U/g (BcL -PVA) and 9.0 U/g (free BcL)	Tóth et al. (2025) [114]
*Burkholderia cepacia* lipase-polyvinyl alcohol (BcL-PVA)	Entrapment	Para-nitrophenyl phosphate solution: 100 μL, 16.5 mM and in 2-propanol BcL solution: 0.1 mg/mL and in 50 mM Tris buffer/0.4% Triton X-100/ 0.1% gum arabic	Temperature 23 °C Humidity: 26%	Voltage: 23 kV Distance: 10 cm Flowrate: 0.5 mL/h	Uniform and smooth structure Average diameter: 241 nm (PVA) and 335 nm (BcL -PVA)	Tóth et al. (2021) [115]
*Saccharomyces cerevisiae* alcohol dehydrogenase (ADH)/oxidized form of nicotinamide adenine dinucleotide (NAD⁺)-PVA	Entrapment	ADH Solution: 1 mg ADH in 1 mL Tris- hydrochloric acid buffer	Temperature 24 °C Humidity: 50%	Voltage: 20 kV Distance: 9 cm Flowrate: 1.8 mL/h	Uniform and smooth structure Average diameter: 760 ± 330 nm	Iitani et al. (2022) [116]
Pepsin-polyvinyl alcohol (Pepsin–PVA)	Entrapment	25% of pepsin concentration	-	-	Bead average diameter: 70–165 nm Enzymatic activity: 96% Optimal condition: pH 3, 1 h of crosslinking and 25% of enzyme concentration Reusability: 25% after 4 cycles Storage stability: >10% up to 10 days	Loredo‐Alejos et al. (2022) [117]
GO_x_—polyurethane/regenerated cellulose/zeolitic imidazolate framework-8 (GO_x_-PU/RC/ZIF-8)	Encapsulation	PU/RC: PU/CA (20 wt%) with 4/1 and 7/3 mass ratios in DMF/ tetrahydrofuran (1:3) and hydrolysed in 0.1 M sodium hydroxide, washed with deionized water, dried overnight GO_x_ -PU/RC/ZIF: 3 g Zinc nitrate and 0.3 g GO_x_ in buffer	-	Voltage: 20 kV Distance: 9 cm Flowrate: 1 mL/h	Smooth structure Average diameter: 880 nm	Li et al. (2022) [118]
Horseradish peroxidase-poly(vinyl chloride) (HRP-PVC)	Encapsulation	HRP solution: 5 mg/mL	Temperature: 25 °C	Voltage: 13.7 kV Distance: 15 cm Flowrate: 1 mL/h	Smooth structure (PVC) and exhibited bulges (HRP-PVC) Average diameters: less than 1 μm, (PVC) and exceed 1.5 μm (HRP-PVC) Immobilization yield: 100% Enzyme loading: 25 μg/mg Activity retention: over 80%	Zdarta et al. (2022) [22]
*Pycnoporus sanguineous CS43* laccase (PsL-CS4)-PVA, PsL-CS43- PVA/SA and PsL-CS43- PVA/chitosan (CS)	Entrapment	PVA (10 wt% 1:1 high and low molecular weight PVA in deionized water PVA/SA: 8wt% PVA/ 3wt% SA in deionized water PVA/CS (60:40): 8wt% PVA/CS in 2% acetic acid PsL-CS43 solution: 1 ml	-	Voltage: 10, 15 and 16 kV Distance: 10, 15 and 30 cm Flow rate: 0.5, 0.6 and 0.3 mL/h	Consistent and bead-free structure (PVA, PVA/SA and PVA/CS) Beaded structure (all entrapped PsL-CS43) Average diameter: 176.96 ± 5.46 nm (PsL-CS43-PVA), 237.60 ± 11.50 nm (PsL-CS43-PVA /SA), 302.18 ± 73.88 nm (PsL-CS43-PVA /CS) Specific activity: 0.1147 ± 0.021 U/mg (PsL-CS43-PVA), 0.0896 ± 0.013 U/mg (PsL-CS43-PVA/SA), 0.1037 ± 0.005 U/mg (PsL-CS43-PVA /CS) Immobilization rate: 95.18% (PsL-CS43-PVA), 96.92% (PsL-CS43-PVA/SA) and 95.52% (PsL-CS43-PVA/CS)	Alvarado-Ramírez et al. (2024) [103]
*Helicoverpa armigera* carboxylesterase (Ha006a)-PVA/CS	Entrapment	PVA/CS (12 wt%/2 wt%): 2.0 g PVA in 10 mL NaOAc buffer/ 0.2 g CS in 10 mL buffer and 10% acetic acid	Temperature: 25 ± 1 °C Humidity: 35 ± 1%	Voltage: 21 kV Distance: 12 cm Flow rate: 0.3 mL/h	Uniform, smooth and bead-free structure (PVA/CS and Ha006a-PVA/CS) Average diameter: 170.5 ± 44.2 nm (PVA/CS) and 222.5 ± 66.5 nm (Ha006a-PVA/CS)	Kaur et al. (2024) [119]

## Optimization parameters in electrospinning and their effects on the properties of entrapped and encapsulated enzyme electrospun nanofibers

The morphology and diameter of entrapped and encapsulated enzyme electrospun nanofibers is affected by three parameters: spinning solution, process, and environmental parameters. Solution parameters such as concentration, molecular weight, chain entanglement, viscosity, surface tension, and solution conductivity significantly impact nanofibers morphology. Ambient parameters like temperature and relative humidity also affect the electrospinning process. Electrospinning parameters include applied voltage, flow rate and the distance between the needle and collector [109, 110]. Tables 1, 2 and 3 summarizes the parameters used in previous studies on entrapped and encapsulated enzyme electrospun nanofibers.

### Solution parameters

Polymer molecular weight, concentrations, solution viscosity, conductivity, surface tension, and solvent selection are the main characteristics that affect an electrospinning process [120–122]. Polymer concentration is a critical parameter as it governs chain entanglement and nanofibers morphology. At low polymer concentrations, insufficient chain entanglement causes the polymer jet to break into fragments before reaching the collector, resulting in beaded nanofibers [123, 124]. The surface tension on electrospun nanofibers morphology depends on the type of solvent used such as ethanol, methyl cellulose, and DMF. The solvent ratio affects both surface tension and solution viscosity, thereby improving the quality and structure of the resulting nanofibers [112].

The viscosity of a solution plays a key role in determining the structure of electrospun nanofibers. When the solution is too diluted and has low viscosity, fiber formation becomes difficult, often resulting in bead-like structures due to insufficient polymer chain entanglement [125]. In contrast, higher viscosity lowers surface tension and promotes the formation of smooth, uniform, bead-free fibers [126]. Viscosity can be controlled by changing the concentrations of the enzyme and polymer in the solution. Giraldi et al. [87] observed that TVL-PLLA nanofibers with 5.1% w/w TVL had a larger diameter (0.81 ± 0.29 μm) compared to the PLLA-Pluronic® F127 control (0.42 ± 0.16 μm), which was attributed to increased solution viscosity. However, in another study, very high enzyme concentrations (0.20–0.25%) have been recorded to disrupted stable electrospinning, producing finer but irregular fibers (451 ± 109 nm), while lower concentrations (0.05–0.10%) ensured stable jet formation and smooth fibers [88]. Overall, enzyme loading influences not only the morphology and diameter of electrospun fibers but also their catalytic performance. Therefore, identifying an optimal enzyme concentration is essential to achieve both stable fiber formation and high enzymatic activity.

An increase in conductivity will lead to smaller electrospun nanofibers diameters [127]. Incorporating bayberry pomace anthocyanin extract increases the solution’s conductivity, resulting in a reduction of electrospun nanofibers diameters by up to 30% [128]. Hosseini et al. [86] reported that diluting an emulsion with EC solution increased conductivity (19 ± 2 to 27 ± 5 μS/cm), improving spinnability. Similarly, Kaur et al. [119] found that PVA/CS nanofibers with higher CS content exhibited increased conductivity (1240 µS/cm), forming the smallest and smoothest fibers (218.5 ± 58.2 nm). This is due to the increased CS concentration, reduced bead formation and improved fiber quality due to hydrogen bonding.

Solvent selection is a critical parameter in the fabrication of smooth and bead-free electrospun nanofibers, as it determines polymer solubility, evaporation rate, and jet stability. High-volatility solvents such as dichloromethane promote rapid evaporation and smooth fiber formation, whereas low-volatility solvents can lead to irregular fibers [129]. The molecular weight of the polymer is also an important solution parameter that affects viscosity, surface tension, conductivity, and dielectric strength [130]. Higher molecular weight increases viscosity, improving fiber formation, while lower molecular weights lead to bead formation [131]. Proper selection of solution parameters is crucial for achieving stable electrospinning and high-quality electrospun nanofibers.

### Ambient parameters

Temperature and relative humidity are critical environmental parameters that must be carefully controlled during the synthesis of electrospun nanofibers. These factors significantly influence fiber morphology, structural integrity, and overall performance, particularly in systems involving enzyme encapsulation or entrapment. Temperature, in particular, affects the electrospinning process by altering solution viscosity, surface tension, and the rate of solvent evaporation. These changes, in turn, impact the formation and physical characteristics of the resulting nanofibers. At lower temperatures, nanofibers tend to form solid cores with smooth surfaces, whereas higher temperatures promote rough surfaces, porous structures, and reduced nanofibers diameters due to faster solvent evaporation and greater jet stretching [123, 132]. For example, De Vrieze et al. [133] investigated the electrospinning of cellulose acetate (CA)/dimethylacetamide and polyvinylpyrrolidone (PVP)/ethanol solutions at varying temperatures (10, 20, and 30 °C). Their results showed that fiber diameter initially increased from 10 to 20 °C but then decreased at 30 °C. This outcome was attributed to reduced viscosity and surface tension at higher temperatures, which enhance polymer stretching. These findings emphasise on the importance of precisely regulating temperature conditions to achieve desirable nanofiber morphology and ensure efficient enzyme immobilization. In systems where enzymatic activity and structural stability are crucial, optimizing environmental parameters is essential for maintaining the functional integrity and performance of electrospun nanofibers.

Elevated humidity slows solvent evaporation and delays jet solidification, which has been associated with the formation of smaller nanofibers with pores or irregular morphologies [21, 134]. In contrast, low humidity accelerates solvent loss, yielding smoother and denser nanofibers, but excessive drying at very low levels can cause brittleness or nanofibers rupture [134]. Drosou et al. [135] demonstrated that humidity levels above 60% led to fused nanofibers while levels below 30% yielded brittle structures in *β*-carotene-loaded nanofibers. Although this study focused on *β*-carotene, the principle is transferable to enzyme encapsulation, where stability demands are even greater. Increasing humidity during electrospinning reduced nanofibers diameter by >10%, with PVA decreasing from 390 to 254 nm and PEO from 394 to 187 nm, highlighting the importance of optimizing electrospinning parameters for achieving uniform and stable electrospun nanofibers [136]. According to Kopp et al. [137], an increase in spinning temperature from 25 °C to 35 °C and humidity from 25 to 30% results in uneven fiber diameters. The most uniform fiber diameters of 998 ± 63 nm were obtained at 30 °C and 25% relative humidity. The influence of these parameters is, however, polymer-dependent. Most studies conducted under controlled conditions (temperature: 20–25 °C, relative humidity: 30–51%) demonstrated bead-free, uniform nanofibers with enhanced enzyme retention and activity (Table 4). Nonetheless, there is a research gap that need to be filled due to the limited number of studies that have thoroughly studying the effect of parameters on the properties of entrapped and encapsulated enzymes electrospun nanofibers.

**Table 4:** Application of encapsulated and entrapped enzymes and free enzymes

Application	Enzyme	Immobilization method	Additional result	Reference
Therapeutics and drug delivery system				
Therapeutic	ALP-PEO	Encapsulation	Encapsulation efficiency: 94.6 ± 5.4% Relative activity: ~100% Enzyme loading: 20.6 ± 1.2%w/w	Onyekuru et al. (2021) [105]
	ALP-PEO	Encapsulation	Encapsulation efficiency: 86.2 ± 2.9% Relative activity: ~100% Enzyme loading: 16.6 ± 0.6%w/w	
Pancreatin replacement therapies	BcL-PVA	Entrapment	Efficiency of p-nitrophenyl palmitate conversion: 74.9% Specific enzyme activity: 137 U/g	Tóth et al. (2021) [115]
Self-sustained antimicrobial	GO_x_/PVA-glucose/PVA	Encapsulation	Antimicrobial activity: No *Escherichia coli (E. coli) and Staphylococcus aureus (S. aureus)* growth after 2 weeks of incubation. Inhibition zones of 20 mm for *E. coli* and 23 mm for *S. aureus* Comparable 1% H₂O₂ and storage stability of ~140 μM after 7 days at 25 °C	Leonarta, & Lee (2021) [106]
Biosensor				
Optical fiber biosensor for smart textile	GO_x_-PVA	Encapsulation	Sensitivity: 1.875 dB mg/ml	Li et al. (2021) [82]
	ADH/NAD⁺-PVA	Entrapment	Detection of ethanol: 2.7–27.3 nmol/s Fluorescence: Nicotinamide adenine dinucleotide (NADH) emission detected (460–490 nm) under UV (365 nm) Optimal condition: pH 8, 20 s and 10 μmol NAD^+^	Iitani et al. (2022) [116]
Biosensor for oxidatino of amines and alcohols and biocatalysis	TVL-PLLA	Encapsulation	Oxidation of catechol: 64% conversion and optimal condition (0.1 mmol of catechol (20 mM), 24 h, acetate buffer and pH4.5) Oxidation of amines: 79% isolated yield, d yield drops from 100 to 22% after 4 cycles and optimal condition (42 mM 1a amine, 2,2,6,6-tetramethylpiperidin-1-oxyl (TEMPO) 20%, acetate buffer and pH 4.5) Oxidation of alcohols: no reaction and optimal condition (83 mM alcohol, TEMPO 20%)	Giraldi et al. (2022) 97]
Smart Textile				
Wearable electronics	GO_x_-PU/RC/ZIF	Encapsulation	Open circuit voltage: 0.35 V Maximum power density: 1.09 W/m^3^ at 0.25 V Voltage remains at 0.32 V under 50% stretching elongation	Li et al. (2022) [118]
Food Industry				
Production of animal food	VuPhy-PVA/SA	Entrapment	Relative activity: 99% pH & thermal stability: higher relative activity across a wide pH & temperature range and greater thermal stability at high temperatures than free VuPhy Optimal condition: pH 6 with 55 °C (VuPhy-PVA) and pH 5 with 45 °C (free VuPhy) Optimal condition: 1.17 mM (VuPhy-PVA) and 0.46 mM (free VuPhy)	Duru Kamaci and Peksel (2021) [113]
Production of hexanal in food industry	LI/LOX/Tween20/NaCl-PGPR	Encapsulation	Production of hexanal: up to 58 mg/L after 92 days Optimal condition: high LI/LOX concentration	Ojstršek et al. (2024) [88]
Environment and Wastewater Treatment				
Removal of persistent sulfamethoxazole and carbamazepine	HRP-PVC	Encapsulation	Removal of SMX and CBZ: both 80% (HRP-PVC) 100% and 75% (free HRP) Optimal condition: pH 7, 25 °C, 24 h, 1 mg/L of pollutants and 2 mM of H_2_O_2_ (HRP-PVC and free HRP) Storage stability: >60% after 20 days at 4 °C (HRP-PVC) and no recycling potential (free HRP) Reusability: >60% after 10 cycles (HRP-PVC) and <20% after 20 days Kinetic parameters: 1.54 mM (free HRP) and 1.8 mM (HRP-PVC) *V*_max_: 422 ± 4 U/mg (free HRP) and 312 ± 20 U/mg (HRP-PVC)	Zdarta et al. (2022) [22]
Removal of 2,4,6-trinitrotoluene (TNT)	PsL-CS43-PVA, PsL-CS43- PVA/SA and PsL-CS43- PVA/CS	Entrapment	Stability: 85.60% at 45 °C and ~100% in the range of pH 2–8 Removal of TNT: 93.43% at pH 7 and 25 °C within four days, preserving 24.13% activity after 5 cycles (PsL-CS43-PVA/SA) Optimal condition: pH 3 and 45 °C (all immobilized PsL-CS43) and pH 2 at 40 °C (free HRP) Storage stability: ~65% (PsL-CS43-PVA), 95.18% (PsL-CS43-PVA/SA) and ~45% (PsL-CS43-PVA/CS) after 4 weeks Reusability: 77.85% (PsL-CS43-PVA/SA) and almost completely lost (PsL-CS43-PVA/CS and PsL-CS43-PVA) after 5 cycles Kinetic parameters: 0.06725 mM (PsL-CS43-PVA/SA) and 0.2480 mM (free PsL-CS43) *V*_max_: 0.3045 mM/s (Ps-CS43-PVA/SA) and 0.3041 mM/s (free PsL-CS43)	Alvarado-Ramírez et al. (2024) [103]
Pesticide bioremediation	Ha006a-PVA/CS	Entrapment	Stability: ~100% at 40 °C (Ha006a-PVA/CS) and ~100% at 35 °C, low activity (5–15 °C), lost function >50 °C (free Ha006a) Active (10–55 °C) and higher relative activity than free Ha006a across a wide temperature range (10–65 °C) Optimal condition: pH 8 and 35 °C (Ha006a-PVA/CS) and pH 7 and 40 °C (free Ha006a) Storage stability: >50% activity after 12 months (Ha006a-PVA/CS) and >50% up to 5 months (free Ha006a) *K*_m_: 012.27 ± 0.238 µM (Ha006a-PVA/CS) *V*_max_: 0.359 ± 0.006 µmoles/min (Ha006a-PVA/CS)	Kaur et al. (2024) [119]

### Processing parameters

The morphology of encapsulated and entrapped enzymes electrospun nanofibers is significantly affected by the distance between the needle and the collector, voltage and flowrate. Onyekuru et al. [105] optimized coaxial electrospinning using core and shell flow rates of 0.1 mL/h and 0.6 mL/h, respectively, at 12 kV and 15 cm, producing stable, bead-free alkaline phosphatase (ALP)-PEO nanofibers. For blend electrospinning, 10 kV voltage, 22.5 cm distance, and 0.8 mL/h flow rate ensured uniform nanofibers formation and controlled solvent evaporation. Optimization of electrospinning parameters directly affected nanofibers morphology and enzymatic activity. Virly et al. [58] stated that a higher flow rate with shorter distance yielded nonhomogeneous nanofibers with low stability, whereas at 0.009 mL/min, 13.0 kV, and 15.0 cm, the process produced smooth, bead-free nanofibers with higher enzymatic activity. These results highlight the importance of optimized conditions for achieving uniform nanofibers and preserving enzyme functionality in encapsulated nanofibers These findings demonstrate that optimized electrospinning conditions are critical for maintaining nanofibers uniformity and enhancing enzyme characteristics in encapsulated enzyme electrospun nanofibers.

Voltage plays a crucial role in determining nanofibers morphology by allowing the polymer jet to overcome surface tension and form a Taylor cone. Increasing the applied voltage generally enhances polymer stretching and electrostatic forces, leading to the formation of smaller nanofibers [138]. However, only within a specific voltage range may electrospun nanofibers develop, as improper voltage levels can cause bead formation. A voltage lower than 10 kV often prevents the formation of a stable jet [139]. An optimal needle to collector distance of 13–15 cm ensures proper fiber stretching and solvent evaporation, resulting in improved electrospun nanofibers morphology. Too short a distance can prevent nanofibers solidification, while excessively long distances may cause bead formation or deposition instability due to incomplete solvent drying, highlighting the need for an optimal distance [110, 140].

A lower flow rate contributes to smoother and more uniform electrospun nanofibers [108, 118]. An increase in flow rate beyond the critical value may result in bead formation and enzymatic activity decreased. For example, Duru Kamaci and Peksel [112] reported that the enzyme activity increased with increasing flow rate up to 0.3 mL/h due to a higher amount of encapsulated *Vigna unguiculata* phytase (VuPhy) in the electrospun nanofibers. However, the biomolecule activity decreased at higher flow rates, probably as a result of the formation of beads in the final electrospun material and easy leaching of enzymes from the electrospun nanofibers.

## Application of entrapped and encapsulated enzymes electrospun nanofibers

Biocatalysis facilitates the precise, prompt, and efficient reduction or degradation of pollutants, transforming them in less harmful or even acceptable forms. Recent studies have demonstrated that entrapped or encapsulated enzyme electrospun nanofibers can effectively serve as biocatalysts. For instance, Koplányi et al. [88] stated that the immobilized *Petroselinum crispum* phenylalanine ammonia lyase (PcPAL) electrospun nanofibers was successfully utilized in the ammonia elimination reaction from L-phenylalanine with the specific biocatalytic activity and specific enzyme activity serving as primary indicators of performance. The PcPAL-PLA nanofibers with 0.15 w/w% enzyme loading exhibited the highest UB (~0.035 U/g) and UE (~30 U/g) which corresponds to the most effective enzyme loading for efficient ammonia elimination. Free PcPAL exhibited a higher UE (~95 U/g) but lacked the advantages of immobilization. The results showed that the PcPAL-PLA biocatalyst is effective in catalyzing the ammonia elimination reaction from L-phenylalanine, though immobilization may introduce diffusion limitations that reduce overall activity.

In organic-phase biocatalysis, *Burkholderia cepacia* Lipase (BcL) immobilized in PVA demonstrated significant potential for enhanced enzymatic activity and reusability. The specific enzymatic activity of BcL-PVA was found to be 90.6 U/g, compared to 9.0 U/g for free BcL, indicating a substantial improvement in performance upon immobilization. BcL-PVA showed no loss in activity after two cycles of reuse, while free BcL exhibited a 30% loss in activity under the same conditions [79]. These studies highlighted the versatility of entrapped and encapsulated enzymes electrospun nanofibers in biocatalysis by providing high enzymatic activity, stability, reusability and efficiency for sustainable applications in green chemistry. They enabled the development of biocatalytic systems with potential applications in therapeutics, drug delivery, biosensors, smart textiles, the food industry, and environmental and wastewater treatment. Table 4 shows a comprehensive review of recent research on entrapped and encapsulated enzymes electrospun nanofibers. This review aims to address research gaps and further the understanding of these biocatalytic systems.

### Therapeutic and drug delivery system

Immobilized enzyme electrospun nanofibers have emerged as a highly promising platform for various therapeutic applications. Tóth et al. [115] stated that pancreatin replacement therapies which aim to restore enzymatic activity in patients with pancreatic insufficiency can benefit from the enzyme such as *Burkholderia cepacia* Lipase (BcL). The entrapment of BcL in PVA demonstrated a significant efficiency in converting p-nitrophenyl palmitate, achieving an impressive 74.9% conversion rate. The specific enzyme activity of the immobilized BcL-PVA system has been reported to be 137 U/g, highlighting its potential for therapeutic applications in enzymatic replacement therapies. Onyekuru et al. [105] reported that alkaline phosphatase (ALP) has been encapsulated in electrospun nanofibers for therapeutic purposes. As reported by Leonarta and Lee [106], the immobilized GO_x_ in the Glu electrospun nanofibers exhibited strong antimicrobial activity against *Escherichia coli* (*E. coli*) and *Staphylococcus aureus* (*S. aureus*) with no microbial growth observed after 2 weeks of incubation. This electrospun nanofibers exhibited inhibition zones of 20 mm (without glucose) and 32.5 mm (with 0.8% glucose) for *E. coli*, and 23 mm (without glucose) for *S. aureus*, highlighting its self-sterilizing capability comparable to 1% H₂O₂. The result demonstrated that the continuous production of H₂O₂ from the immobilized GO_x_ electrospun nanofibers effectively prevents microbial growth, highlighting its potential for antimicrobial applications. These findings reinforced the feasibility of immobilized enzyme electrospun nanofibers for therapeutic applications, including enzyme replacement therapy, antimicrobial coatings, and biocatalysis.

### Biosensor

Biosensors are advanced analytical devices that provide real-time, highly sensitive, and selective detection of biomolecules, making them essential for applications in clinical diagnostics, food safety, and environmental monitoring [82]. One promising method in biosensor development involves the use of entrapped and encapsulated enzymes electrospun nanofibers. Iitani et al. [116] highlighted the viability of using immobilized ADH electrospun nanofibers as ethanol gas sensors to detect ethanol gas in the range of 2.7–27.3 nmol/s over 20 s. Nicotinamide adenine dinucleotide (NADH) absorption at 340 nm indicated that enzyme activity is maintained. Fluorescence measurements at 365 nm UV irradiation detected NADH emission at 460–490 nm, supporting the enzymatic conversion of ethanol. With a 79% isolated yield in amine oxidation under ideal conditions (42 mM 1a, 2,2,6,6-tetramethylpiperidin-1-oxyl (TEMPO) 20%, acetate buffer pH 4.5), TVL-PLLA electrospun nanofibers with 3.2%w/w enzyme loading showed great promise for biosensor applications in the determination of amines. Its yield dropped from 100 to 22% during four cycles, indicating a considerable decrease in recyclability, most likely as a result of enzyme deactivation [87]. The glucose sensor sensitivity of 1.875 dB/mg. mL was achieved by encapsulating GO_x_ in PVA [82]. These studies demonstrate the potential of entrapped and encapsulated enzymes electrospun nanofibers for biosensing applications, including ethanol gas detection, amine oxidation, and glucose sensing, with high sensitivity and efficiency, though recyclability remains a challenge.

### Smart textile

Smart textiles enable various functions such as energy harvesting and storage, drug release and optics [141]. However, conventional energy storage options, such lithium-ion batteries, are inflexible and need to be recharged frequently. This underscores the necessity of adaptable, environmentally friendly, and sustainable power sources. Enzymatic biofuel cells which employ immobilized enzymes to convert chemical energy in electricity, present a promising solution. This solution provides continuous, self-sustained power directly from the body or environment. Enzyme immobilization in textile fibers enables efficient energy harvesting, providing sustainable power for wearable electronics while ensuring biocompatibility and stability for long-term health monitoring [142]. One notable advancement in this field is the encapsulation of laccase and GO_x_ enzymes in PU/RC membranes, with the addition of a Metal–Organic Framework such as zeolitic imidazolate framework-8 (ZIF). It is possible to create stretchy enzymatic biofuel cells using electrospun nanofibers. The system demonstrates promising performance with an open circuit voltage of 0.35 V and a maximum power density of 1.09 W/m^3^ at 0.25 V. Even during 50% stretching elongation, the voltage remained constant at 0.32 V. The study demonstrated the robustness and flexibility of the electrochemical system for usage in smart textile [118].

### Food industry

Recent work by Duru Kamacia and Peksel [113] demonstrated the successful entrapment of *Vigna unguiculata* phytase (VuPhy) in PVA/SA nanofibers, resulting in enhanced enzymatic properties. A slow release of enzyme is more favourable in food applications as it ensures prolonged enzymatic activity and stability. The VuPhy-PVA/SA nanofibers exhibited a high relative activity (99%) and superior pH and thermal stability compared to free VuPhy. Specifically, immobilized phytase maintained activity across a broader pH and temperature range, demonstrating greater stability at high temperatures. The optimal conditions for VuPhy-PVA were pH 6 at 55 °C, whereas free phytase performed best at pH 5 and 45 °C. The Michaelis–Menten constant (*K*_m_) of immobilized phytase was 1.17 mM, significantly higher than the 0.46 mM of free VuPhy, indicating improved substrate affinity and enzyme performance. These findings highlight the potential of entrapped VuPhy electrospun nanofibers for animal feed applications and food preservation, ensuring sustained enzyme activity for extended periods. Ojstršek et al. [88] also reported that the immobilized LI/LOX electrospun nanofibers enabled sustained hexanal release (up to 55.8 mg/L at 25 °C and 53 mg/L at 1 °C) over 92 days and showed promising applications in extending the shelf life of fresh fruits and vegetables.

### Environment and wastewater treatment

In recent years, numerous industries have released enormous amounts of biologically toxic chemicals in water bodies, causing severe water pollution. These industrial chemicals include organic compounds, inorganic compounds and oils. Approximately 220 billion tons of chemicals are released annually from various industries [143]. Industries such as refining [144], textile industry [145], dairy industry [146], pharmaceuticals [147], leather industry [148], and pulp and paper industry [149] are significant contributors, generating vast amounts of wastewater laden with pollutants. The improper treatment of industrial chemicals disrupts environmental balance, human health, and water quality, leading to water scarcity. This challenge aligns with United Nations Sustainable Development Goal 6, which focuses on ensuring access to clean water and sanitation, which underscores critical issues of addressing water pollution, especially as over a billion people are projected to face water shortages by 2025 [150]. To address these challenges, enzymatic wastewater treatment which utilizes enzymes as biocatalysts has been developed to remove or degrade wastewater pollutants [68].

Enzymatic wastewater treatment refers to a promising approach for high-specificity pollutants degradation with minimum environmental impacts [151]. Over the years, enzymatic wastewater treatment approaches have evolved to address the limitations of slow growth-dependent bioremediation and to reduce or even remove toxic pollutants. Compared to traditional chemical and biological treatments, this technology provides enhanced and specific degradation capabilities, eliminates the challenges of shock loading or startup/shutdown delays typically associated with plant operations, and delivers improved efficacy in targeting specific compounds of interest [152]. Enzymes such as lipase, laccase, and peroxidase are widely used commercially due to their ability to oxidize a broad range of pollutants in wastewater [153]. Enzymes are also smaller in size compared to microbial cells, enabling them to easily come in contact with pollutants and exhibit faster mobility.

Immobilized enzymes can be environmental pollution control in industry [154]. Zdarta and colleagues [22] have demonstrated the feasibility of immobilized laccase for environmental applications, with HRP proving highly effective in removing pharmaceutical contaminants. The removal efficiency of SMX and CBZ by immobilized HRP exceeds 80%, whereas free HRP achieved 100% removal of SMX and 75% removal of CBZ over a 24-h period. Pollutant concentration, pH, temperature, H₂O₂ concentration and enzyme loading all play a crucial role in determining removal efficiency. The optimal conditions for the degradation of SMX and CBZ by both free and immobilized HRP were found to be pH 7, a temperature of 25 °C, a reaction time of 24 h, and an initial pollutant concentration of 1 mg/L and an H₂O₂ concentration of 2 mM.

Alvarado-Ramírez et al. [103] recorded the utilization of nanofibers for the removal of 2,4,6-trinitrotoluene (TNT) in wastewater treatment. Entrapped *Pycnoporus sanguineous* laccase *CS43* (PsL-CS43) in PVA/SA electrospun nanofibers exhibited excellent TNT biodegradation by achieving 93.43% removal within 4 days and retained 24.13% of its catalytic activity after 5 consecutive cycles. Additionally, it retained 85.60% of its activity at 45 °C and nearly 100% stability in the pH range of 2–8. This high biotransformation efficiency across multiple cycles, excellent thermal and pH stability highlights the practical applicability of immobilized laccase in pollutant degradation. Electrospun nanofibers also show strong potential for pesticide bioremediation. For example, Kaur et al. [119] demonstrated that *Helicoverpa armigera* carboxylesterase (Ha006a) electrospun nanofibers retained approximately 100% stability at 40 °C, and remained active across a wide temperature range (10–55 °C). In contrast, the free Ha006a enzyme maintained similar stability only up to 35 °C, as it exhibited low activity between 5 and 15 °C and lost functionality above 50 °C. Subsequently, the immobilized enzyme not only demonstrated broader thermal stability, but also exhibited higher catalytic efficiency, positioning Ha006a-PVA/CS electrospun nanofibers as a promising candidate for pesticide and environmental bioremediation.

## Challenges and future directions

One of the most critical challenges in electrospinning lies in optimizing the interplay of process parameters, including polymer concentration, applied voltage, needle-to-collector distance, flow rate and temperature. Although these operational factors can be controlled, environmental fluctuations frequently lead to bead formation and inconsistency, ultimately compromising the quality of electrospun products. Conventional electrospinning setups also suffer from low throughput, batch-to-batch variability, and inconsistent nanofibers morphology. These drawbacks pose significant barriers to industrial-scale applications, especially for enzyme immobilization. Maintaining enzyme stability during the electrospinning process further complicates scale-up. In addition, gradual enzyme leaching reduces catalytic efficiency in long-term applications, undermining the reusability of these systems. To date, most electrospun nanofibers have primarily served either as enzyme supports or filtration membranes. This singularity of function restricts their potential for broader, multifunctional applications. A major challenge lies in selecting carrier polymers that are not only biocompatible with the enzymes but also capable of forming stable nanofibers without obstructing active sites. Moreover, unresolved toxicological and physicochemical concerns surrounding nanomaterials, coupled with limited understanding of mechanisms such as active site blocking, hinder their broader adoption. Improving enzyme longevity and performance remains a key challenge for translation to practical implementations [155].

Looking forward, the development of advanced electrospinning systems with regulated environmental conditions will be essential to improving reproducibility and fiber uniformity. Future research should focus on scalable electrospinning systems capable of producing nanofibers with well-controlled diameter and surface properties, while retaining enzyme activity and function. Economic feasibility is another critical aspect. The incorporation of cost-effective carriers and eco-friendly solvents will be essential to ensure economic feasibility or transitioning from lab-scale experimentation to real-world applications. Ensuring long-term operational stability is equally important to ensure practical deployment. Strategies such as polymer crosslinking, surface modification, and incorporation of stabilizers should be further optimized to mitigate enzyme leaching and activity loss over time. These advancements must be complemented with systematic studies under operational conditions (pH, ionic strength, temperature, and flow dynamics). Such studies, paired with detailed structural and mechanistic analyses, will provide insights necessary for designing durable and efficient nanofiber-based biocatalysts.

Integration into continuous bioreactors represents a particularly promising avenue, enabling nanofibers to function as both catalytic supports and filtration units. This dual-functionality enhances process efficiency and opens new avenues for real-time water purification and biochemical synthesis. Additionally, co-entrapment or co-encapsulation of multiple enzymes represents an exciting frontier to achieve synergistic catalysis. Such systems can mimic natural metabolic pathways, enabling sequential or cascade reactions that significantly improve efficiency in pollutant degradation and biosensing. These multifunctional platforms have the potential to revolutionize biocatalysis by combining high performance with operational simplicity. To fully unlock this potential, in-depth mechanistic studies are required to elucidate polymer–enzyme interactions and enzyme orientation to design carriers that maximize activity. The adoption of advanced characterization techniques, such as molecular simulations, can provide critical insights into the accessibility of enzyme active sites and the influence of polymer matrices on enzyme function. Finally, comprehensive toxicological assessments must be conducted to ensure the environmental and biological safety of nanofiber-based systems, particularly for applications involving direct human or ecological exposure. Addressing these challenges will be essential for advancing electrospun nanofibers to scalable, safe, and sustainable biocatalytic platforms.

## Conclusion

Electrospun nanofibers incorporating encapsulated or entrapped enzymes offer considerable advantages over free enzymes, particularly in terms of enhanced catalytic activity, improved stability, and reusability. The structural versatility of electrospun nanofibers allows for the fine-tuning of fiber morphology, porosity, and surface properties, enabling efficient enzyme immobilization and substrate interaction. These features have enabled applications in bioremediation, water treatment, therapeutics, antimicrobial materials, and smart textiles development. Despite challenges in process optimization, scalability, enzyme stability, and mechanistic understanding, ongoing advances in electrospinning design, polymer selection, and multi-enzyme co-encapsulation are steadily addressing these limitations. These innovations point toward the creation of integrated, high-performance biocatalytic platforms. The continued development of interdisciplinary research will be key to translating these systems into efficient, sustainable, and multifunctional biocatalytic platforms. Systematic studies of enzyme–polymer interactions, supported by advanced characterization tools and toxicological assessments, are essential to ensure the safe and effective deployment of these systems. In conclusion, with continued innovation and cross-disciplinary research, enzyme-loaded electrospun nanofibers hold strong promise as scalable, sustainable, and multifunctional platforms for industrial, biomedical, and environmental applications.

## Data Availability

No datasets were generated or analysed during the current study.
